# Gelatin Stabilizes Nebulized Proteins in Pulmonary Drug Delivery against COVID-19

**DOI:** 10.1021/acsbiomaterials.2c00419

**Published:** 2022-05-24

**Authors:** Chunlin Li, Ira Marton, Daniel Harari, Maya Shemesh, Vyacheslav Kalchenko, Michal Pardo, Gideon Schreiber, Yinon Rudich

**Affiliations:** † Department of Earth and Planetary Sciences, Weizmann Institute of Science, Rehovot 76100, Israel; ‡ Department of Biomolecular Sciences, Weizmann Institute of Science, Rehovot 76100, Israel; § Department of Veterinary Resources, Weizmann Institute of Science, Rehovot 76100, Israel

**Keywords:** RBD-62, pulmonary delivery, gelatin stabilization, CoV-19

## Abstract

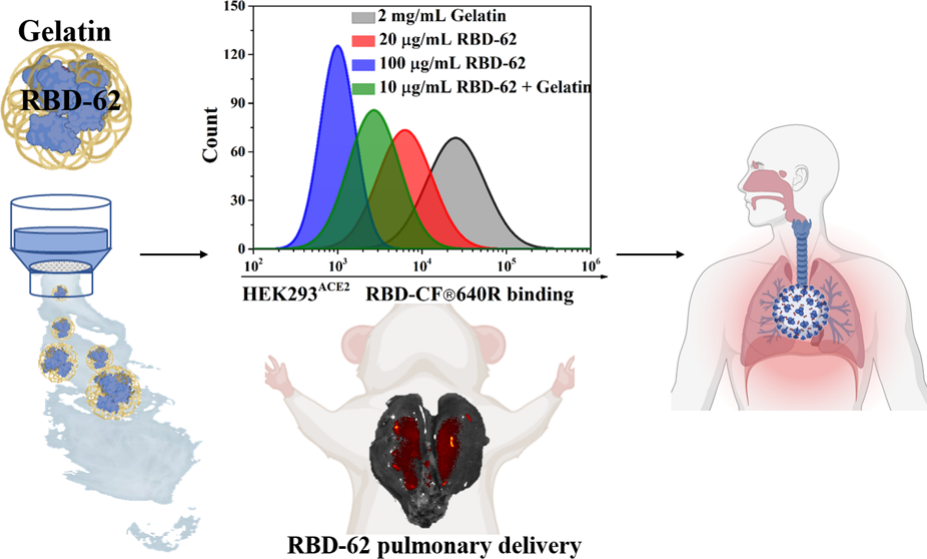

Delivering medication to the lungs via nebulization of pharmaceuticals is a noninvasive and efficient therapy route, particularly for respiratory diseases. The recent worldwide severe acute respiratory syndrome coronavirus type 2 (SARS-CoV-2) pandemic urges the development of such therapies as an effective alternative to vaccines. The main difficulties in using inhalation therapy are the development of effective medicine and methods to stabilize the biological molecules and transfer them to the lungs efficiently following nebulization. We have developed a high-affinity angiotensin-converting enzyme 2 (ACE2) receptor-binding domain (RBD-62) that can be used as a medication to inhibit infection with SARS-CoV-2 and its variants. In this study, we established a nebulization protocol for drug delivery by inhalation using two commercial vibrating mesh (VM) nebulizers (Aerogen Solo and PARI eFlow) that generate similar mist size distribution in a size range that allows efficient deposition in the small respiratory airway. In a series of experiments, we show the high activity of RBD-62, interferon-α2 (IFN-α2), and other proteins following nebulization. The addition of gelatin significantly stabilizes the proteins and enhances the fractions of active proteins after nebulization, minimizing the medication dosage. Furthermore, hamster inhalation experiments verified the feasibility of the protocol in pulmonary drug delivery. In short, the gelatin-modified RBD-62 formulation in coordination with VM nebulizer can be used as a therapy to cure SARS-CoV-2.

## Background

Inhalation of nebulized medications represents an effective pulmonary delivery method for healing because of the high bioavailability of the drugs via the noninvasive route to the lungs.^1,2^ The other motivation of pulmonary drug delivery is to treat respiratory diseases like asthma, pulmonary fibrosis, chronic obstructive pulmonary disease (COPD), etc.^3^ Direct access to the site of disease allows for a high local concentration of active pharmaceutical ingredient (API), thereby minimizing medication dosage and increasing their effectiveness and safety.^4^ As such, various peptides and proteins are under development for treating lung diseases or for pulmonary vaccination.^5^ For example, a measles vaccination administered by inhalation was reported to be superior to a parenteral vaccination and IFN-β has been evaluated for curing COVID-19 through inhalation.^6−8^

The worldwide COVID-19 pandemic resulted in the death of millions of people from infection, with more enduring secondary impacts.^9,10^ Severe acute respiratory syndrome coronavirus type 2 (SARS-CoV-2) virus and its variants recognize cells by binding their spike protein with high affinity to the angiotensin-converting enzyme 2 (ACE2) in the lung cells through its receptor-binding domain (RBD). A series of vaccines have been developed to fight the infection, including revisionary mRNA vaccines coding for the SARS-CoV-2 spike protein.^11^ However, due to the high rate of virus mutations, the virus became partially resistant to vaccines, particularly at the RBD, where most neutralizing antibodies bind. Moreover, these same mutations drastically reduced the efficacy of most of the neutralizing antibodies given as drugs to more severely infected patients.^12^ This calls for the continuous development of updated vaccines or medicines that are efficient, easy to administer, and resilient to viral mutations. As reported in our previous work, the developed high-affinity variant RBD-62 can be used as a drug to inhibit infection with SARS-CoV-2 and associated variants in vitro and in vivo.^13^ Pulmonary delivery of RBD-62 via nebulization provides a promising alternative pathway to protect the public.

A known problem in the delivery of biological drugs (proteins and nucleic acids) by nebulization is their susceptibility to the stress encountered during the process.^14^ This can result in their degradation, inactivation, adsorption, unfolding, and/or aggregation due to the large air–liquid interface in the micron-sized droplets, a challenging problem in many applications of sensitive proteins in pulmonary drug delivery.^15^ Several solutions have been proposed, including better design of nebulizers and formulation development, to obtain stable proteins for the nebulization.^16^ Vibrating mesh (VM) nebulizers provide advantages in medication efficiency, nebulization rate, and reproducibility over other types, e.g., jet and ultrasonic nebulizers.^17−19^ In addition, VM nebulizers can rule out aerosol recirculation and hold consistent solvent concentrations during operation.^20,21^ The specific design of VM nebulizers (such as the PARI eFlow) reduces reservoir heating and likely eliminates thermal degradation of the protein during the operation.^15^

Apart from the nebulizer’s design for small molecular drugs, excipients, including sugars, polyols, etc., are used to formulate biopharmaceuticals considering their viscosity, surface tension, ionic strength, and pH that can affect biomolecular activity.^22,23^ Polysorbate 80 and 20 surfactants (PS80, PS20), for example, are incorporated in formulation nebulization to protect sensitive proteins at the air–liquid interface.^24^ Nevertheless, according to the Food and Drug Administration (FDA, https://www.fda.gov/), the range of additive ingredients currently developed and approved for pulmonary delivery is very limited. One such additive is gelatin, a water-soluble polypeptide with many natural sources. It has been widely used as a carrier in drug delivery and for controlled drug release because of its biocompatibility and security, and also versatile features in pharmaceutical and medical applications.^25^ Moreover, its presence has been shown to stabilize various proteins by increasing their melting temperature and reducing aggregation.^26^

Here, we investigated the nebulization of RBD-62, IFN-α2, and bovine serum albumin (BSA) proteins using two types of VM nebulizers, Aerogen Solo and PARI eFlow. After evaluating several protein supplements, gelatin was selected as a good excipient to improve the formulation for better stabilization. The effect of gelatin on droplet size, recovery, and biological activity was investigated, and optimized gelatin concentration in the formulation was determined. Moreover, pulmonary delivery of the aerosolized formulation containing gelatin and RBD-62 was conducted on hamsters, showing stable RBD-62 binding throughout the lungs. The results verified the effectiveness of gelatin in stabilizing all three proteins, even at low concentrations, resulting in recovery above 80%. The data provided here rationalize the use of RBD-62 as a strategy and therapy that can help protect against COVID-19.

## Materials and Methods

**Figure 1:**
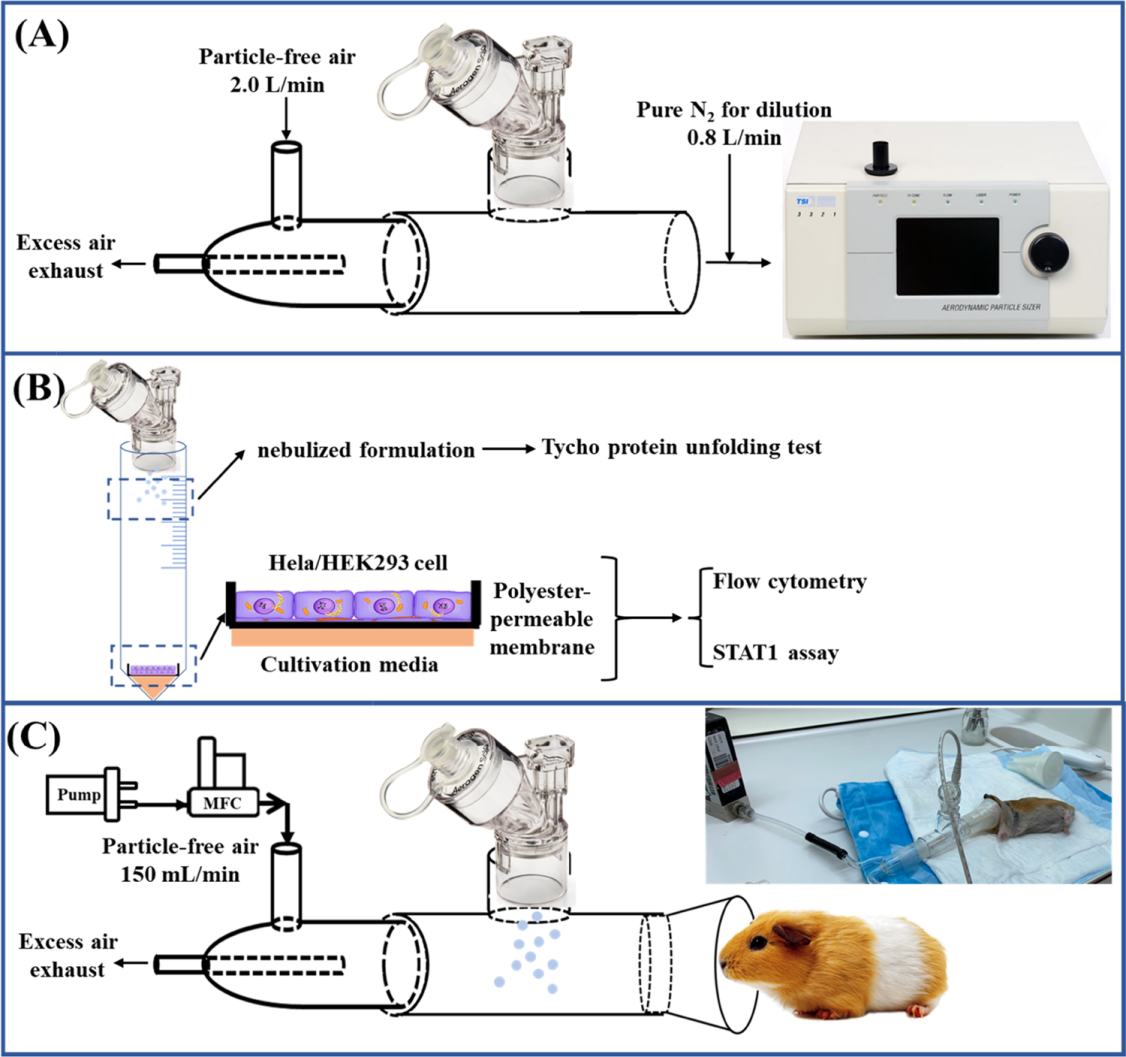
Method and experimental setup including (A) nebulizer test for operation rate and size distribution measurements of generated droplets via self-designed T-impinger connector and aerodynamic particle sizer (Model 3321, TSI). (B) Cell exposure setup for generating protein droplets and the method for protein collection and activity tests. For clarity, only aerogen nebulizer-connected setup is presented. (C) Hamster exposure setup for protein nebulization.

The whole experimental setup and contents are displayed in Figure 1, including evaluation of VM nebulizer operation in protein-droplet generation (Figure 1A), cellular (Figure 1B), and animal (Figure 1C) test of nebulized proteins.

### Protein Recovery Measurement Using Tycho NT6

The production of IFN-α2 and RBD-62 was described referring to Piehler and Schreiber^27^ and Zahradník et al.,^13^ respectively. Capillary tubes filled with pre- and postnebulized protein solutions were inserted into channels of the Tycho NT6 instrument (NanoTemper Technologies). Each sample’s fluorescence is recorded upon temperature increase from 35 to 95 °C. The brightness for proteins at different concentrations was used to construct a linear calibration curve via eq 1
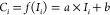
1where *f*(*I*_*i*_) is the specific linear function connecting concentration, *C*_*i*_, of a sole untreated protein *i* and the detected brightness, *I*_*i*_, and *a* and *b* are parameters for the linear function.

Based on the calibration functions constructed for various proteins, the recovery, *R*_*i*_, for protein *i* after nebulization can be derived according to eq 2
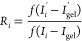
2

In eq 2, *I*_*i*_ and *I*_*i*_’ are brightness for protein *i* before and after nebulization, respectively, and *I*_gel_ and *I*_gel_’ are brightness for gelatin before and after nebulization, respectively. If without gelatin, *I*_gel_ and *I*_gel_’ are zero.

### Binding Competition

HEK293^ACE2^ cells overexpressing ACE2 (HEK293/ACE2 stable cell line, GenScript Cat. No. M00770) were seeded (1 × 10^5^ in 0.5 mL of DMEM at 37° with 95% air and 5% CO_2_) on a 12-well plate or a 12 mm diameter Transwell 0.4 μm Pore Polyester Membrane Insert (insert) (Corning, Inc. Transwell Cat No. 3460). Each insert occupies a well in a 12-well plate with 1–1.5 mL of medium below and 0.5 mL above the insert membrane. On the following day, the inserts were transferred to a 50 mL conic “falcon” tube containing 0.5 mL of medium. The medium attached to the insert’s membrane from below forms an air–liquid interface. We used the vibrating mesh nebulizer (VMN) Aerogen Solo (Aerogen, Inc.) to nebulize protein formulation (see Figure 1B). The RBD-62 solution (5–100 μg/mL) in 0.2 mL/insert of 0.5× PBS as is or with 2 mg/mL gelatin (Merck, Inc., Sigma-Aldrich G8150). And 15 min after exposure to RBD-62 droplets, 100 μL of medium was added to cover the cells on the membrane. Then, 6 μL of WT-RBD labeled with CF640R Succinimidyl Ester added to the medium (labeling of 20 mM RBD by Biotium, Inc., Cat. #92108) was performed at 60 mM in 0.1 M bicarbonate for 1 h incubation under gentle shaking. Then, the protein was dialyzed before use. After 1 h incubation at 37 °C, the cells were removed from the insert by EDTA (0.05 M in PBS), diluted, and washed twice with PBS + 0.5% BSA to remove toxic EDTA and unbound labeled WT-RBD. The cells were analyzed by flow cytometry (Amnis CellStream Flow Cytometer, Luminex) to measure the difference in fluorescence intensity (Excitation at 642 nm, Emission at 702/78) between cells treated with nebulized blank protein (2 mg/mL gelatin) and cells treated with RBD-62 (5–100 μg/mL), with more than 4500 cells analyzed/treatment at the final gate.

### Phospho-Flow Cytometry Measurements for STAT1

HeLa cells were seeded at a density of 10^5^ cells/0.5 mL DMEM on Corning, Inc. Transwell Cat No. 3460. This 12 mm Transwell has a 0.4 μm Pore Polyester Membrane Insert (insert). And 24 h after seeding, the insert with the cells was transferred from the Transwell to a 50 mL conic “falcon” tube containing 0.5 mL of medium. The medium attached to the insert’s membrane from below forms an air–liquid interface. Cells were treated with nebulized IFN-α2 or IFN-β (6 μg + 0.1 mg of gelatin in 200 μL of PBS solution, see Figure 1B) directly via deposition of droplets. As a negative control, a HeLa cell line with knockout for both the interferon receptors (IFNAR1 and IFNAR2) was used^28^ and treated with the same nebulized IFN-α2 or IFN-β. After IFN nebulization (induction), 0.1 mL of media was added for an additional 45 min. Then, the cells were detached from the insert membranes by Trypsin EDTA solution (Biological Industries, Cat. #03–050–1B). After incubation, the media with trypsin was replaced. Harvested cells from two inserts were combined as one treatment. The cells were twice washed in 0.5% BSA in PBS. Next, the cells were fixed by adding a solution of 2% paraformaldehyde (PFA) in PBS and incubated at RT for 15 min. Then, PFA was removed by centrifugation for 5 min at 1*g*, and pellets were washed once in 0.5% BSA in PBS. Permeabilization: Cell pellets were resuspended in 0.5 mL of ice-cold methanol and incubated at −20 °C for 30 min. Then, the tube was filled with wash buffer, and the cells were centrifuged for 7 min at 1*g* and then washed two more times. The pellet was resuspended in 50 μL of anti-phosphorylated pSTAT1 (Tyr.701) monoclonal antibodies (BD Bioscience Alexa Fluor 647 Mouse Anti-Stat1 (pY701) cat. #BD612597) diluted by 3/100 μL in wash buffer. The cells were incubated for 1 h and then washed twice before flow cytometry analysis. Five repeats analyzing 1000–7000 cells at the final gate were measured upon excitation using the 642 (red) laser and emission at 702/78 nm.

### Hamster Inhalation

Golden Syrian Hamsters (*Melanochromis auratus*, Jackson Laboratories), age 8–10 weeks, weighing 115–125 g, were anesthetized by Ketamine and Xylazine injection (IP) and kept on warm heating mats during their period of sedation. RBD-62 labeled with CF640R at the indicated doses was solubilized in the described doses in eluent (1.5 mL 0.5× Ca/Mg-free PBS + 2 mg/mL gelatin). The animals were subjected to nebulization using the self-assembled drug delivery apparatus (Aerogen nebulizer and air supply system) with an airflow of 0.15 L/min. A conical mask was generated by a three-dimensional (3D) printer to fit to the nebulizer. Conical-shaped masks for the hamsters were designed by 3D printing, using Shapr3D CAD software (Sharpr3D, Budapest, Hungary). The printing process utilized IdeaMaker 3D slicer software and Raise3D-Pro2 printer from (Raise3D, Irvine, CA). This 3D printer is based on fused deposition modeling (FDM), which falls under the material extrusion category of the 3D printing technology, using polylactic acid (PLA) filaments for the printing process. A description of the experimental setup is presented in Figure 1C.

The conical opening of each mask was covered with a layer of parafilm, after which a hole poked through the center to allow for direct flow of nebulized drug to the hamsters’ nose and mouth, with minimal loss of material around the sides of the face. Hamsters were allowed to fully recover, with no signs of distress detected for the animals. We noted condensation of drugs within the nebulization chamber. Of the 1.5 mL of the nebulized material, ∼50% (∼0.75 mL) was lost to condensation for each animal. By 30 min post-nebulization, all animals had recovered from anesthesia. At the end timepoint (as indicated), the animals were injected with a lethal dose of Pentobarbital, after which their lungs were extracted and rinsed in PBS, before being fixed in 4% paraformaldehyde-PBS (PFA-PBS) for 2 days before being replaced with 1% PFA-PBS for a week (4 °C). The fixed tissues were then placed in an IVIS spectrum (IVIS Spectrum In Vivo Imaging System from PerkinElmer), and fluorescence was measured with Excitation-640 nm/Emission-680 nm and an exposure time of 10 s. Selected lung samples were then placed in 30% Sucrose/PBS, placed in OCT blocks before generating 10 μm sections using a Leica CM1950 cryostat. Slides were images with a Leica Mi8 microscope equipped with a motorized stage and a Leica DFC365 FX camera. Experiments were performed with animal ethical committee’s guidance: Weizmann IACUC #01740221-2.

## Results

Proteins were nebulized to form micron-sized droplets from aqueous formulations. Two types of vibrating mesh nebulizers (5.0 μm porosity), Aerogen Solo and PARI eFlow, were used for protein nebulization. Both nebulizers are readily available and approved for medical inhalation purposes. These portable devices work efficiently with small volumes of liquids and have minimal dead volume loss. An aerodynamic particle sizer (APS, Model 3221, TSI) was used to characterize the size distribution of the droplets immediately generated from these nebulizers (Figure 1A). The mean PBS droplet aerodynamic size was 1.96 and 1.45 μm from Aerogen and PARI nebulizers, respectively (Figure S1A). Both are thus suitable for drug delivery into the lung by inhalation, where particles of radii of 1–5 μm are optimal. Next, we measured the nebulization rates of both devices (Figure S1B). The nebulization time linearly correlated with the loaded volume for Aerogen type but best fits a logit model, with solution volume added to the PARI nebulizer due to its specific design. The output flow is vertical for the Aerogen nebulizer, while the flow is horizontal for the PARI nebulizer. It is noted that the PARI solution reservoir is up to 6 mL but with a dead volume of 0.6 mL. The nebulization rate of Aerogen is the smaller of the two devices, with a volume of up to 2 mL nebulized in 250 s, while the PARI holds 6 mL of solution, nebulized in 400 s.

### IFN-α2 and RBD-62 Protein Nebulization

Protein nebulization is notoriously difficult due to the marginal thermostability.^15^ Here, two proteins were nebulized using the Aerogen and PARI devices, and the fractions of active proteins in droplets were evaluated (see the Materials and Methods Section). One protein is IFN-α2, a 165-amino-acid-long cytokine that binds to the type I interferon receptors (IFNAR1 and IFNAR2), leading to their dimerization, which drives the activation of the Janus kinase (JAK) and signals transducer and activator of transcription (STAT) signaling cascade (Schreiber, 2017). The second protein is an engineered version of the receptor-binding domain (RBD) of the SARS-CoV-2 spike protein. This protein (called RBD-62) of 193 amino acids has enhanced thermostability and ACE2 binding affinity relative to the RBD of the Wuhan variant.^13^ Unless otherwise stated, all proteins were prepared in phosphate-buffered saline solution (PBS, pH 7.2–7.4). To measure both proteins’ folded state and concentration after nebulization, we used the Tycho NT6 (NanoTemper Technologies), which measures the tryptophan fluorescence of the protein with excitation at 280 nm and emission at 330 and 350 nm. Tryptophan fluorescence is quenched in the folded state and undergoes a redshift upon unfolding. The protein melting temperature (*T*_m_) and its brightness thus provide measures of the protein folded state and its relative concentration. Figure S2 presents the relation between brightness as determined by the Tycho NT6 and protein concentration for the four proteins used in this study (IFN-α2, RBD-62, BSA, and gelatin), with a linear relationship observed for them all.

**Figure 2:**
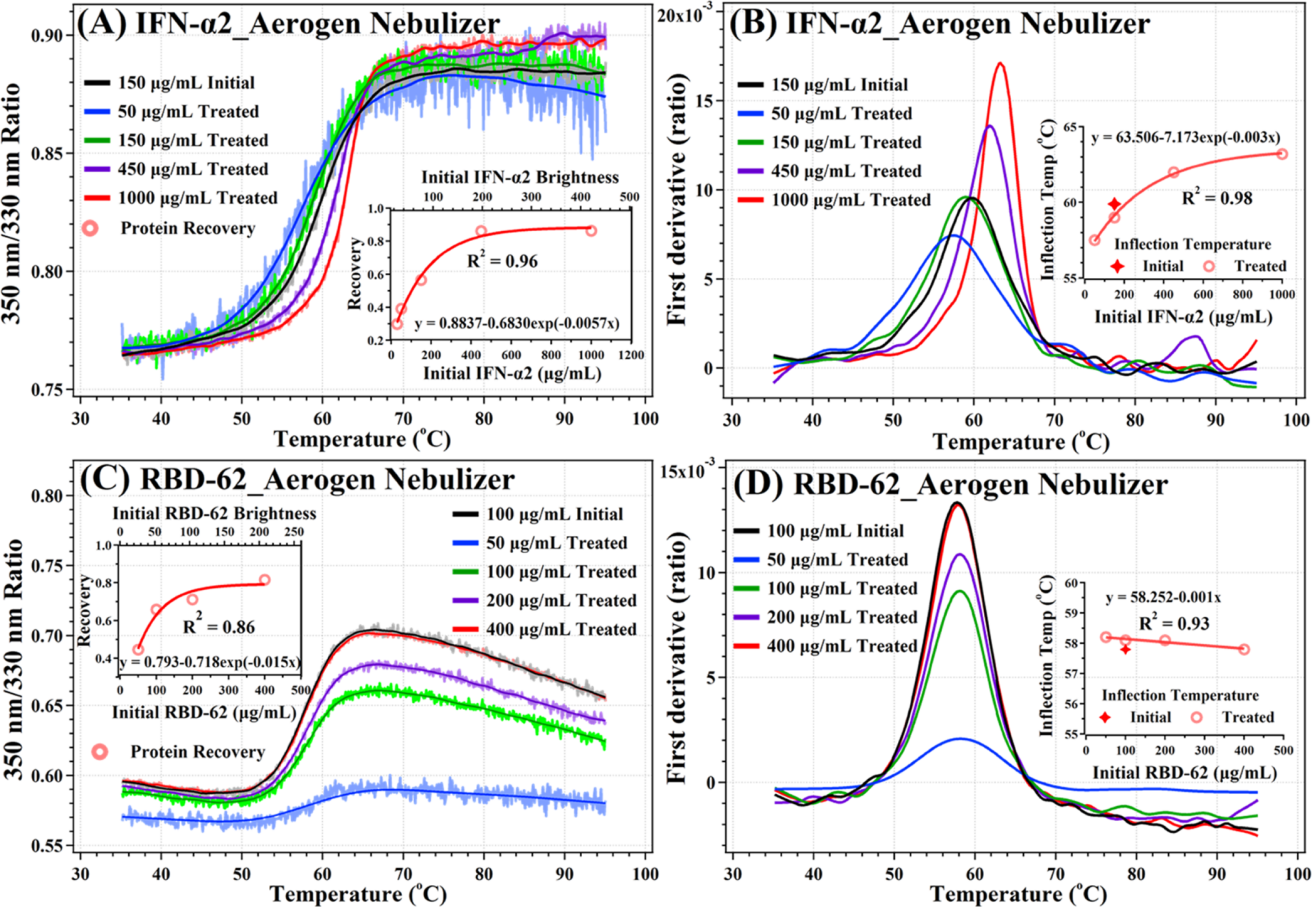
(A, B) Folding profiles for untreated and Aerogen-nebulized IFN-α2 in PBS at various concentrations. (C, D) Folding profiles for untreated and Aerogen-nebulized RBD-62 in PBS at various concentrations. Active protein recovery and inflection temperatures as a function of initial protein concentration are displayed in the inserts of each graph.

Figure 2A,C shows unfolding profiles in terms of the ratio of fluorescence emission intensity at 350 and 330 nm (E350/E330) upon heating for untreated and nebulized proteins at a range of concentrations. IFN-α2 and RBD-62 (0.2 mL) were nebulized using the Aerogen nebulizer and collected in a 50 mL polypropylene tube, as schematically shown in Figure 1B. Then, the protein concentration and folded state were determined using the Tycho NT6. The recovery of the nebulized proteins as a function of input concentration (50–1000 μg/mL) is shown as an insert in Figure 2A (for recovery calculations, see the Materials and Methods section). An ascending exponential decay mode was observed with increasing concentration for the nebulized IFN-α2 recovery. Specifically, the percent recovery was ∼90% at the highest concentration, while only ∼30% at the lowest concentration. The *T*_m_ increased exponentially from 58 to 63 °C with increasing input protein concentrations (Figure 2B). This is attributed to the concentration-dependent dimerization of IFN-α2, which increases its thermostability.^29^ Next, we repeated the same experiments using RBD-62 (Figure 2C,D) with 50–400 μg/mL input concentrations. The fraction recovery displays the same increasing behavior as that of IFN-α2. At the lowest concentration, the RBD-62 recovery was ∼45%, while the recovery exceeded 80% for the highest concentration.

**Figure 3:**
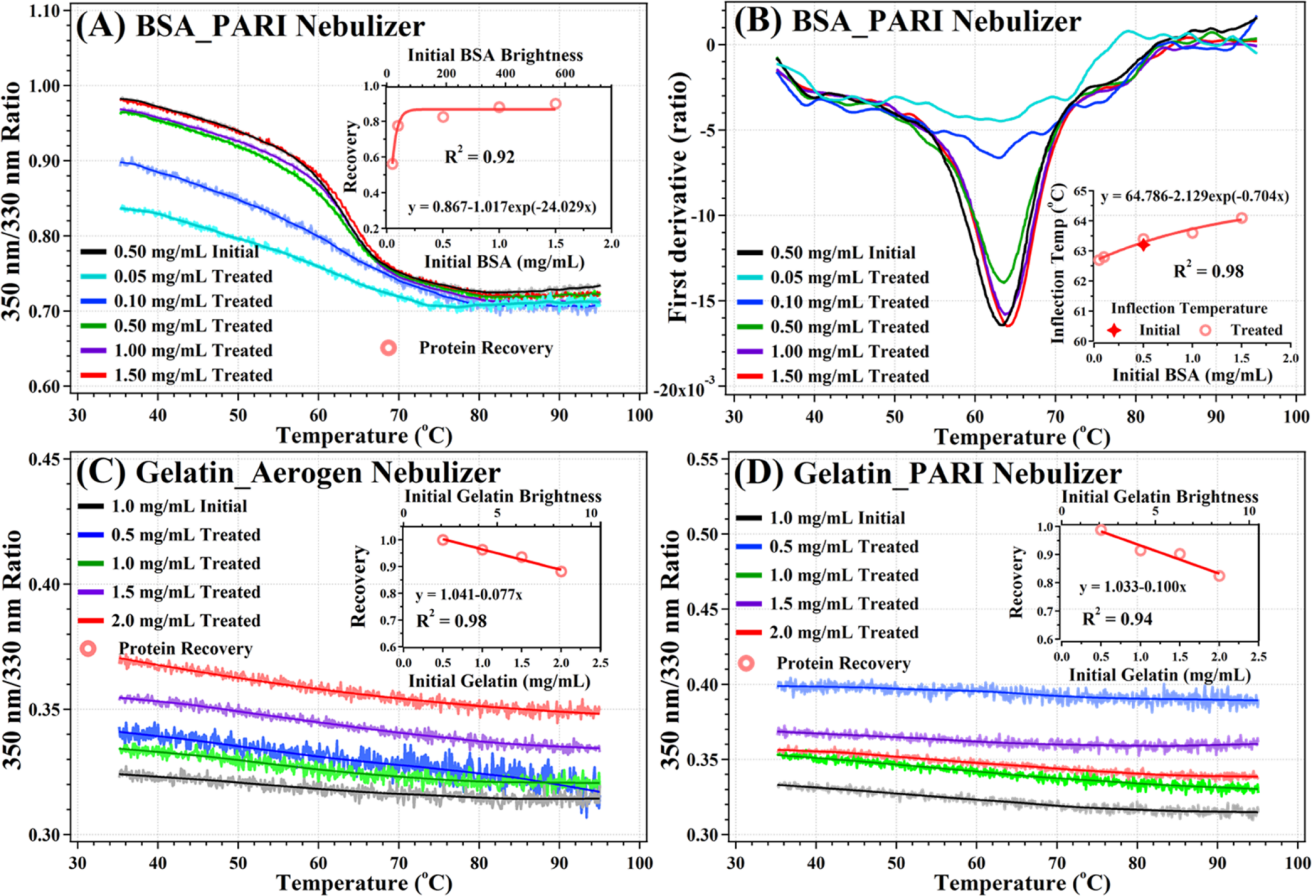
(A, B) Folding profile for the untreated and PARI-nebulized BSA in PBS at various concentrations. (C, D) Folding profile for untreated and Aerogen-nebulized Gelatin in PBS at various concentrations. Active protein recovery and inflection temperatures as a function of initial protein concentration are displayed in the inserts of each figure.

In contrast, with IFN-α2, the *T*_m_ of RBD-62 was not affected by the input concentration, as RBD-62 is a monomer at these concentrations. The experiments were also repeated with the PARI nebulizer for BSA and gelatin proteins (Figure 3). Similar to Aerogen-nebulized IFN-α2, PARI-nebulized BSA also presents increasing recovery along with input concentration (Figure 3A). In contrast, the *T*_m_ increased only slightly at higher concentrations (Figure 3B), possibly due to the tendency of BSA to dimerize.^29^ Gelatin alone did not show significant temperature-dependent signals in the Tycho NT6 (Figure 3C,D). This is because gelatin is a natively unfolded polypeptide and thus has no transition unfolding.

### Using Gelatin to Enhance Protein Recovery during Nebulization

**Figure 4:**
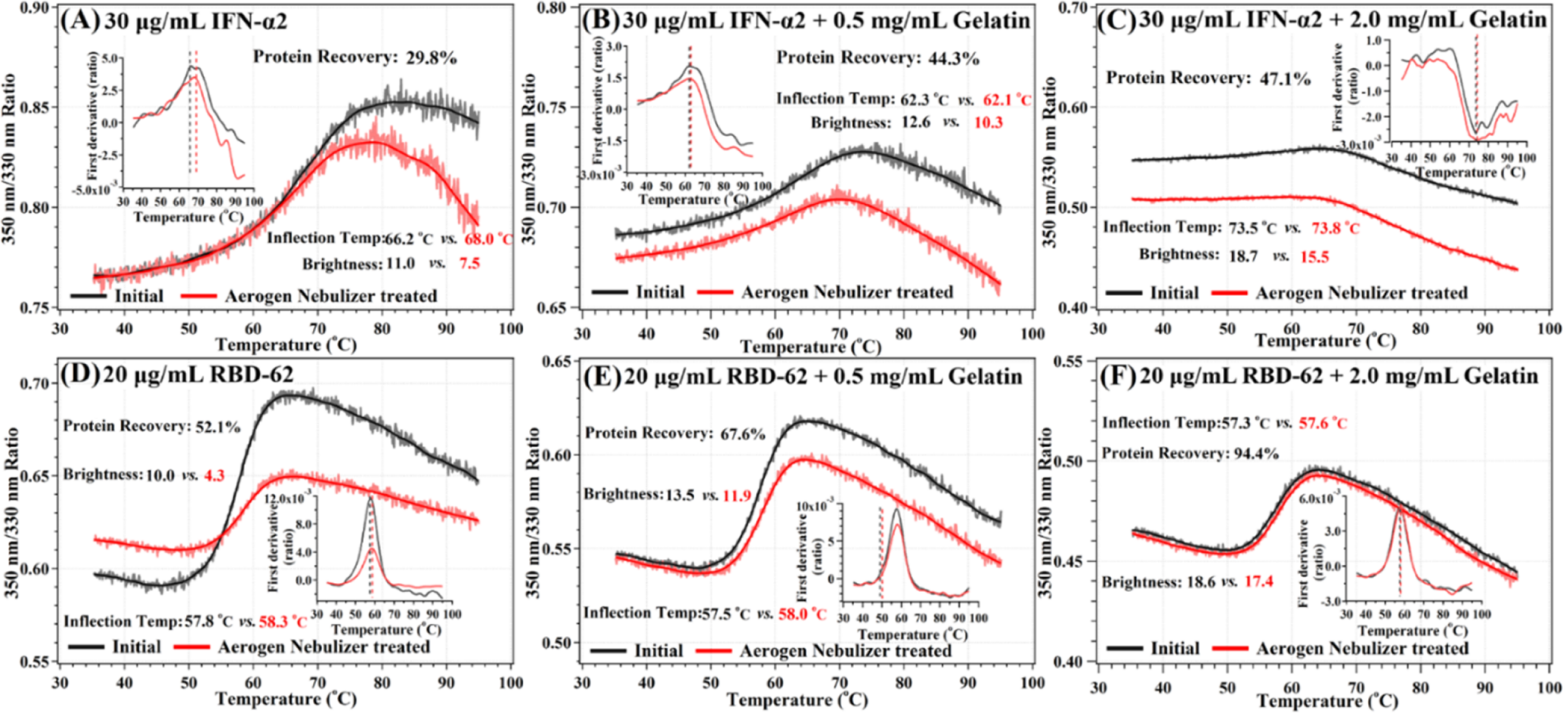
(A–C) Effect of adding gelatin to IFN-α2 folding and recovery following Aerogen nebulization. (D–F) Effect of adding gelatin to RBD-62 folding and recovery following Aerogen nebulization. Insets indicate first derivative of protein unfolding with temperature.

**Figure 5:**
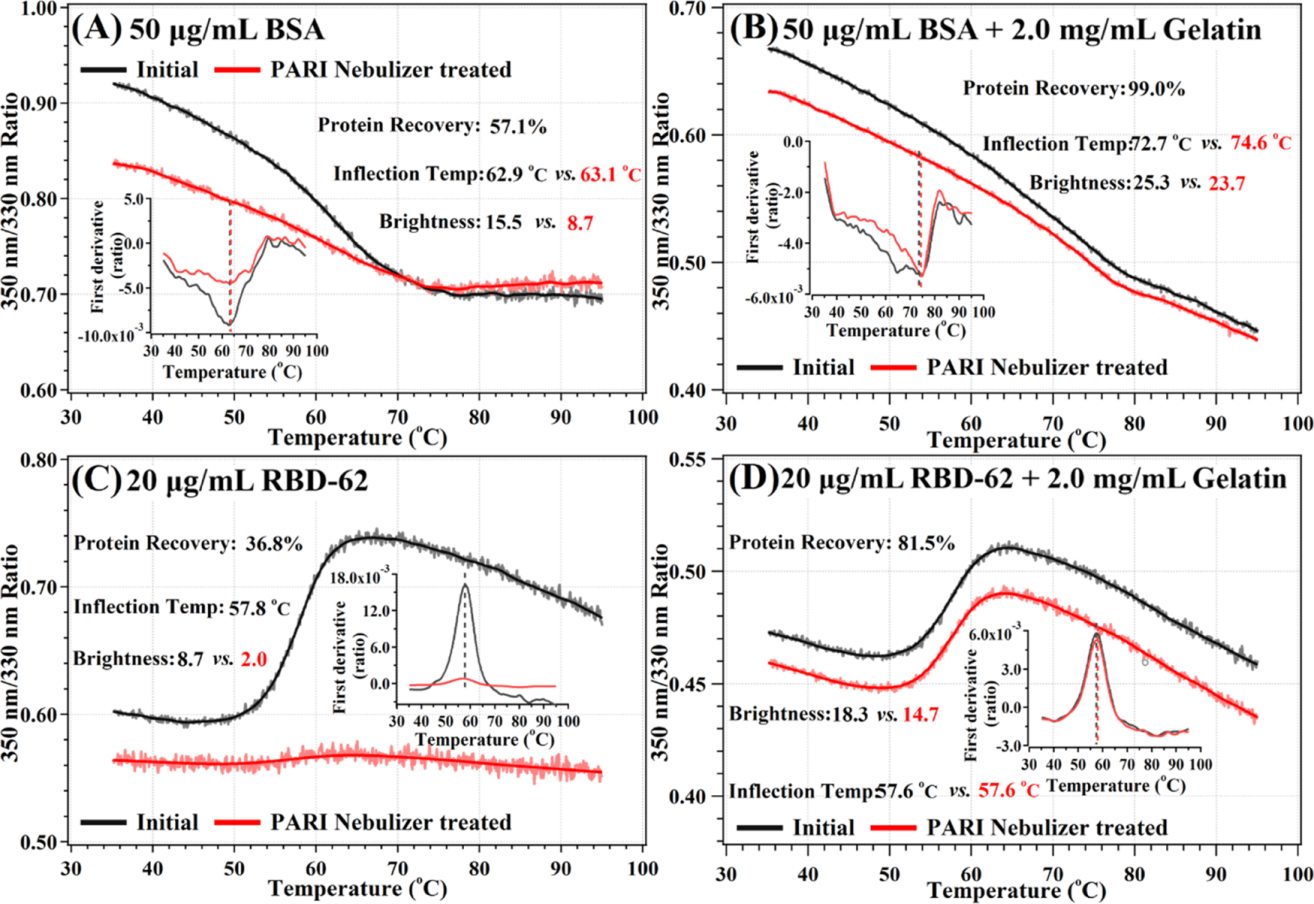
(A, B) Effect of adding gelatin to BSA folding and recovery following PARI nebulization. (C, D) Effect of adding gelatin to RBD-62 folding and recovery following PARI nebulization. Insets indicate first derivative of protein unfolding with temperature.

To enable efficient protein nebulization, we tested the effect of gelatin on the percent recovery of IFN-α2 and RBD-62 at low protein concentrations. In Figure 4A–C, 30 μg/mL IFN-α2 was mixed with 0.5 or 2 mg/mL gelatin and subjected to nebulization using the Aerogen device (0.2 mL input). The gelatin increased percent recovery from 29.8 to 47.1% (see also Table 1). Using the same setup but nebulizing RBD-62 at 20 μg/mL increased recovery from 52 to 94.4% due to gelatin addition (Figure 4D–F). The same experiment was repeated using the PERI nebulizer to test the effect of 2 mg/mL gelatin on protein recovery. Here, the percent recovery of BSA (50 μg/mL, 2 ml input) increased from 57.1 to 99% (Figure 5A,B, and Table 1). The percent recovery of RBD-62 (20 μg/mL, 2 mL input) increased from 36.8 to 81.5% (Figure 5C,D and Table 1). The results clearly show that gelatin stabilizes various proteins (IFN-α2, RBD-62, and BSA) upon nebulization.

**Table 1:** Summary of Gelatin Enhancement in Protein Residence during Nebulization

**protein**				
type	concentration (μg/mL)	**gelatin** (mg/mL)	**nebulizer**	**protein recovery (%)**
IFN-α2	30		Aerogen Solo	29.8
IFN-α2	30	0.5	Aerogen Solo	44.3
IFN-α2	30	2.0	Aerogen Solo	47.1
BSA	50		PARI	57.1
BSA	50	2.0	PARI	99.0
RBD-62	20		Aerogen Solo	52.1
RBD-62	20	0.5	Aerogen Solo	67.6
RBD-62	20	2.0	Aerogen Solo	94.4
RBD-62	20		PARI	36.8
RBD-62	20	2.0	PARI	81.5

### Evaluating the Bioactivity of Interferons after In Vitro Nebulization

**Figure 6:**
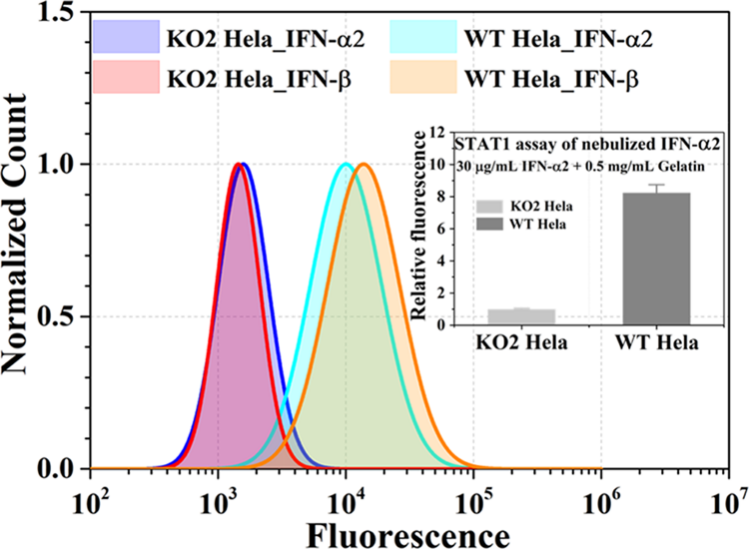
Phosphorylation of STAT1 was measured using phospho-flow cytometry after HeLa cells or HeLa cells with double knockout of the interferon receptors (KO2) were treated with nebulized (Aerogen nebulizer) IFN-α2 or IFN-β (6 μg in 200 μL) for 15 min. The main panel shows the flow cytometry histograms. The inset shows the relative difference in mean fluorescence of wild-type (WT) cells compared with KO2 cells. The mean fluorescence values are shown in Table S1.

Apart from percent recovery, bioactivity is the crucial index to weigh the efficacy of nebulized proteins. The binding of interferons to their receptors results in the phosphorylation of STAT1 proteins that drive gene induction.^30^ We monitored STAT1 phosphorylation in the cells following nebulization of two type I interferon subtypes, IFN-α2 and IFN-β. HeLa cells were used to follow STAT1 phosphorylation. While HeLa cells originated from cervical cancer, their type 1 interferon system is identical to that in any other cell. A schematic view of the setup used for this experiment is shown in Figure 1B. Nebulization was done using the Aerogen nebulizer, with 30 μg/mL (0.2 mL) of either interferon in the presence of 0.5 mg/mL of gelatin. HeLa cells with a knockout of both IFNAR1 and IFNAR2 receptors (KO2)^28^ were used as a negative control. STAT1 phosphorylation was measured using phospho-flow cytometry analysis (for details, see the Materials and Methods Section). Figure 6 shows the induction of phosphorylation upon subjecting HeLa cells to nebulized IFNs. It is clear that interferon is biologically active after nebulization, with no difference seen between using IFN-α2 or IFN-β (fluorescence data in Table S1).

### Evaluating the Bioactivity of RBD-62 after In Vitro Nebulization

**Figure 7:**
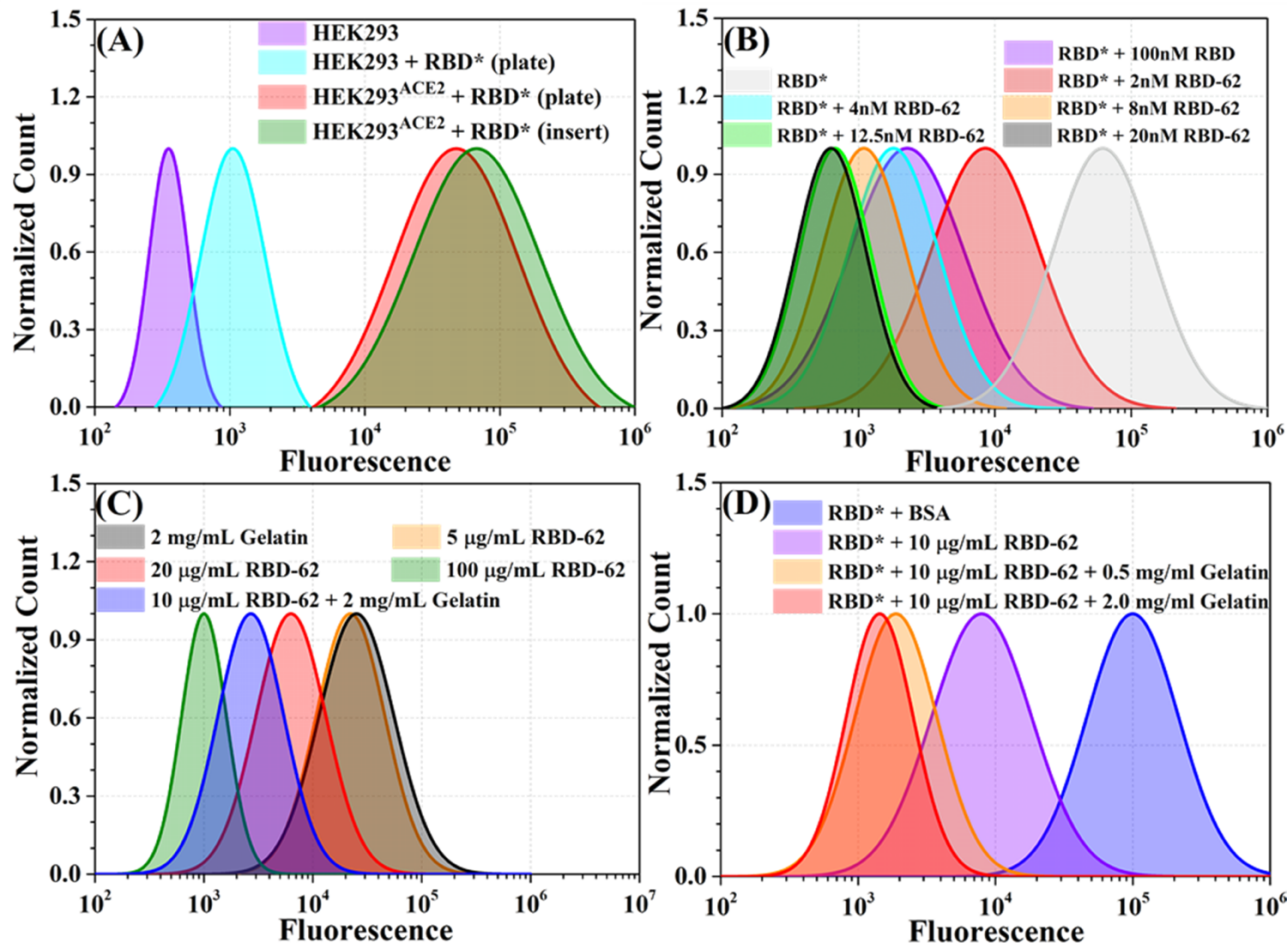
Binding competition of labeled RBD (RBD*) by nonlabeled RBD-62. (A) HEK293 cells or HEK293 cells stably transfected with ACE2 (HEK293^ACE2^) were treated with RBD*, using either a standard plate or an insert that was used also for nebulization. Binding of RBD* leads to higher cell fluorescence as measured using flow cytometry. (B) HEK293^ACE2^ cells labeled with RBD*. The cells were exposed to RBD-62 addition at the indicated concentrations, and cell fluorescence was measured using flow cytometry. (C) HEK293^ACE2^ cells labeled with RBD*. The cells were exposed to nebulized RBD-62 (200 μL) at different concentrations, with or without gelatin (see the Materials and Methods Section and Figure 1B). (D) HEK293^ACE2^ cells labeled with RBD*. The cells were exposed to 200 μL of nebulized RBD-62 at different concentrations, with or without increasing concentrations of gelatin. Fluorescence of the cells was determined by flow cytometry.

RBD-62 is tightly binding to ACE2. To evaluate RBD-62 binding after nebulization, we used HEK293 cells stably transfected with ACE2 (HEK293^ACE2^). Here, HEK293^ACE2^ was used as a model system for cells highly expressing ACE2 so that inhibition of RBD binding to the cell surface could be monitored. First, wild-type RBD (Wuhan variant) was labeled with the fluorescent marker CF640R. The binding of RBD-CF640R to HEK293^ACE2^ cells results in a substantial increase in their fluorescence relative to binding to HEK293, as detected by flow cytometry analysis (Figure 7A). The increased fluorescence was independent of whether RBD-CF640R binding was done on HEK293 cells grown in a standard plate or on an insert used for nebulization (see Figure 1B for the experimental setup). Next, we applied nonlabeled RBD-62 at concentrations from 2 to 20 nM to compete with labeled RBD-CF640R. As seen in Figure 7B, 2 nM RBD-62 inhibited 90% of the RBD-CF640R binding, while complete inhibition is observed at the higher RBD-62 concentrations (left shift in *x*-axis). For comparison, 100 nM nonlabeled RBD-WT reduced RBD-CF640R binding to less than 4 nM RBD-62, showing the effectiveness of RBD-62 to block RBD binding and, as a consequence, virus infection.

**Figure 8:**
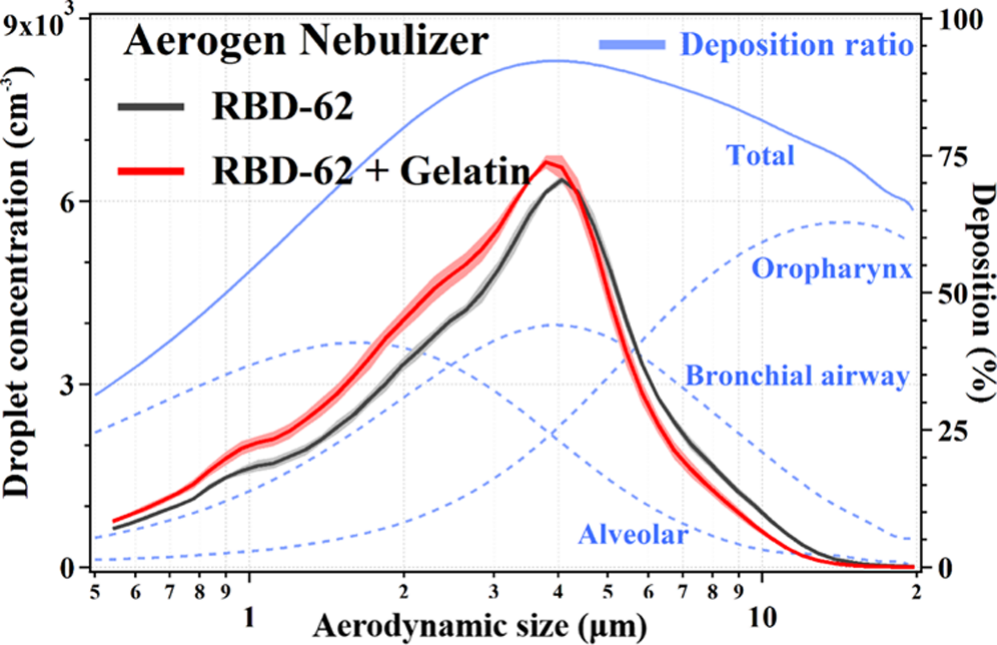
Droplet size-resolved concentrations of Aerogen nebulizer generated RBD-62 vs RBD-62+Gelatin. Background blue dashed and solid lines describe size-dependent deposition ratio of aerosols at different respiratory regions for a normal adult male.

We repeated the competition experiment to establish that the binding competition assay provides quantitative information on RBD binding to ACE2 in a tissue culture setup, including for HEK293ACE2 cells grown on an insert for exposure to nebulized mist now using nebulized RBD-62 (Figure 7C,D). Increasing the concentration of RBD-62 from 5 to 100 μg/mL (0.2 mL input, using the Aerogen nebulizer) increased the inhibition of RBD-CF640R binding, reaching >99% at the highest concentration. Adding 2 mg/mL of gelatin to RBD-62 strongly enhanced its activity after nebulization, with 10 μg/mL + gelatin equivalent to ∼50 μg/mL of RBD-62 alone in inhibiting RBD-CF640R binding (Figure 7C and Table S2). To determine the optimal amount of gelatin, we repeated the experiment, using 10 μg/mL RBD-62 without and with 0.5 and 2 mg/mL gelatin (0.2 mL input, using the Aerogen nebulizer). Clearly, the gelatin enhanced the activity of nebulized RBD-62, with 2 mg/mL gelatin supporting the highest RBD-62 activity (Figure 7D and Table S2). Higher concentrations of gelatin (>2 mg/mL) clogged the nebulizer and thus were not used in this study. Finally, we evaluated the droplet size distributions of nebulized RBD-62 in the presence and absence of gelatin using the Aerogen nebulizer. Gelatin addition slightly decreased the droplet size of nebulized RBD-62, with a mean aerodynamic size of 3.8 μm. The droplet size distribution allows for depositions of the droplets along the respiratory airway, including in alveolar cells (Figure 8). The RBD-62 competition experiment was repeated using the PARI device for nebulization (Figure S3 and Table S3). The experiment was done as in Figure 7C (using the Aerogen device); however, due to the larger dead volume (∼0.6 mL) of the PARI nebulizer, we used 1 mL of solution for nebulization. RBD-62 (30 μg/mL) resulted in complete elimination of RBD-CF640R binding, similar to that observed using the Aerogen device.

### Inhalation of RBD-62 by Hamsters

**Figure 9:**
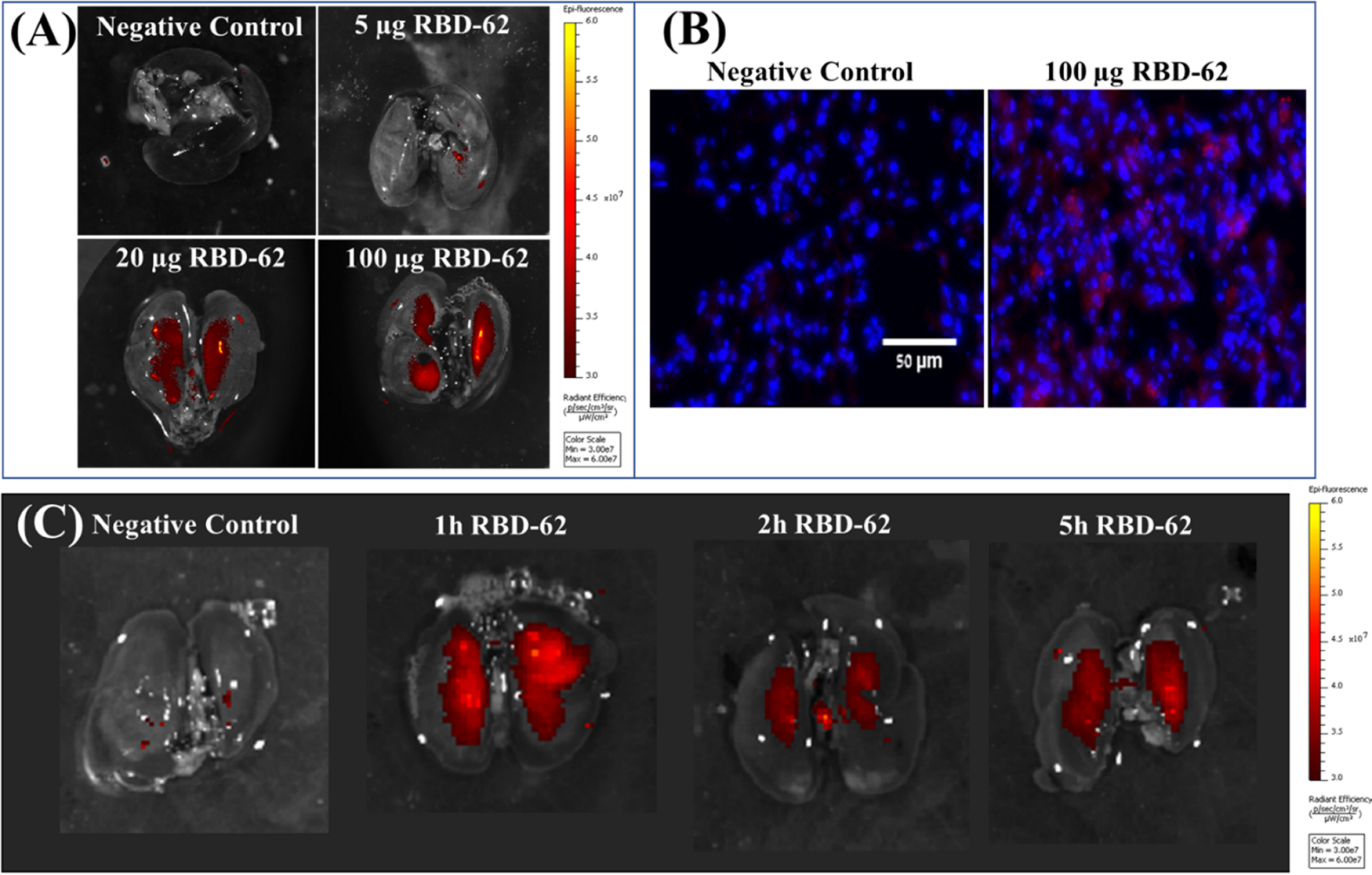
Hamster lungs following nebulization with RBD-62 labeled with CF640R. Animals were nebulized with RBD-62-CR^640 nM^ using a constant volume of eluent (1.5 mL 0.5× PBS + 2 mg/mL gelatin) per animal.** **(A) Dose response of RBD-62, after which the animals were sacrificed 1 h post-drug treatment. (B) Cryosections of two lungs from the dose–response study, detected by fluorescence microscopy. RBD-62-CR^640 nM^ (red) and the nuclear DNA stain DAPI (blue). (C) Time course of 50 μg of RBD-62-CR^640 nM^ in Syrian Hamsters, which were sacrificed at 1, 2, and 5 h post nebulization. In (A) and (C), drug-conjugated fluorescence was measured from extracted lungs using an IVIS optical imaging device. The background-subtracted fluorescence values are in Table S4.

Our aim in establishing nebulization protocols for proteins was to use it as a treatment option. Hamster has been shown as a preferable model for SARS-CoV-2 binding to ACE2, as the binding affinity is similar to that observed to human ACE2, while binding to mice ACE2 is poor.^13^ RBD-62 was specifically engineered to block ACE2 and thereby inhibit SARS-CoV-2 infection. Therefore, we devised a setup for hamster inhalation of RBD-62. The schematic setup of the experiment is shown in Figure 1C, with the inset showing the actual setup. A mini-pump introduced particle-free air directly through a mass flow controller (MFC, MKS Instruments, Inc.) with a flow of 150 mL/min. The airflow carried the nebulized droplets from the Aerogen nebulizer at the center of the T-tube to the side where the hamster was fixed with only its nose and mouth inserted into the tube. The hamsters were briefly anesthetized before the experiment. Based on the hamsters’ weight of about 120 g, a 75–100 mL/min respiration rate was estimated.^31,32^ We applied sufficient air supply to keep a positive pressure throughout the exposure system in the case of contamination from the outside and to ensure that the hamster inhaled air was loaded with droplets from the nebulizer. The excess puff-like air/mist exhausted from the impinger head verified and monitored the hamster breath pattern (see Supporting Video). RBD-62 was labeled with CF640R at doses of 0, 5, 20, and 100 μg added to 1.5 mL eluent per animal, after which the Aerogen nebulizer was activated for 5 min. The nebulization process was typically completed within the first 2–3 min. One hour after the inhalation, the hamsters were sacrificed and the lungs were removed and monitored for accumulation of red color, which indicates RBD-62 binding. As shown in Figure 9A, an explicit dependency of dose versus signal was observed, with the stronger signal noted for higher doses, especially for 20 and 100 μg.

The lungs were further processed for cryo-sectioning and fluorescence histochemistry. Lung sections from the negative control experiments as well as 100 μg of RBD-62-treated animals were sectioned and counterstained with DAPI to allow nuclei visualization (Figure 9B). Fluorescently labeled RBD-62 is seen to surround some of the nuclei (blue) in lung cells from the drug-treated animal only, but not all lung cells. This is consistent with the ACE2 immunohistochemistry pattern of human lungs, where high levels of ACE2 were detected in type II pneumocytes but not for other lung cell types.^33^ Finally, we performed a time course of RBD-62 binding to the Hamster lung over three time points. In this experiment, the animals were nebulized with 50 μg of RBD-62, after which tissues were collected at 1, 2, and 5 h post-drug therapy (Figure 9C). The results show the presence of the RBD signal at all time points, including 5 h post-nebulization, indicating the persistence of the drug in the hamster lungs over time.

## Discussion

The Aerogen and PARI vibrating mesh nebulizers were successfully used for generating micron-sized droplets from protein formulations. We found that proteins such as IFN-α2, RBD-62, and BSA, alone retain relatively low activity in droplets aerosolized by either Aerogen or PARI nebulizer. The addition of gelatin, even at a low concentration (0.5 mg/mL), significantly increases the fraction recovery of nebulized proteins. For example, the addition of 0.5 and 2.0 mg/mL gelatin to 20 μg/mL of RBD-62 increased the protein percent recovery from 52 to 68 and to 94%, respectively, when mixed in formulation. Thus, the loss of protein in the formulation in the two nebulization devices is not due to the reduced concentration of active protein, but only due to the dead volume of the instrument. As mentioned, the Aerogen device has no dead volume, but allows for only 2 mL of solution, which may not be sufficient. The PARI solution reservoir is up to 6 mL but with a dead volume of 0.6 mL. This would suggest using the PARI only when larger volumes are needed.

Moreover, gelatin enhances the bioactivity of nebulized proteins. Specifically, the potency of nebulized RBD-62 at a low concentration to compete with labeled RBD-CF640R was greatly enhanced when mixed with 2 mg/mL of gelatin. As gelatin was shown to be safe to use in inhalation, its protection as a carrier protein for different proteins suggests its use to minimize protein dosage in formulation preparation.

The hamster inhalation experiments verified the feasibility of our nebulization protocol, showing the binding of RBD-62 to lung cells, with the binding being maintained over time. This protocol provides an option for pulmonary drug delivery to outcompete SARS-CoV-2 infection, as shown by Zahradník et al.^13^

In summary, we show here an optimal method for the inhalation of protein drugs, achieving a very high active yield for a number of different proteins. The biggest advantage of drug inhalation is the specific administration to the lungs, which is a main entry port for pathogens and toxic materials. This largely reduces unwanted side effects from systemic administration. While small molecules are always more desirable than proteins as drugs, the recent 20 years have shown that for many conditions, protein drugs are more specific and powerful, and their availability for inhalation extends their scope of use specifically in fighting lung diseases.

## Supporting Information Available

Normalized droplet size distributions for PBS generated by the Aerogen and PARI nebulizer, nebulization time as a function of uploaded PBS solution volume for Aerogen and PARI nebulizer (Figure S1); calibration curve for protein quantification using a Tycho NT.6, relating concentration to protein signal brightness (Figure S2); binding competition of labeled RBD (RBD*) by nonlabeled nebulized RBD-62 + 2 mg/mL Gelatin using the PARI device (Figure S3); mediant fluorescence signals as supplementary of Figure 6 (Table S1); median fluorescence as supplementary to Figure 7 (Table S2); median fluorescence as supplementary to Figure S3; and median fluorescence of Hamster lungs after inhalation of RBD-62 labeled with CF640R as supplementary to Figure 9A and C (Table S4) (PDF)
Supplementary video of Hamster exposure to nebulized RBD-62 (MP4)
