# Supplementary material for: Gelatin Stabilizes Nebulized Proteins in Pulmonary Drug Delivery against COVID-19
Source: ACS Biomater Sci Eng. 2022 May 24;8(6):2553–63. doi: 10.1021/acsbiomaterials.2c00419 (PMC9159517; doi:10.1021/acsbiomaterials.2c00419)
Supplement: Supplementary file 1 — ab2c00419_si_001.pdf [file ab2c00419_si_001.pdf]

**Supporting Information**

# Gelatin stabilizes nebulized proteins in pulmonary drug delivery against COVID-19

Chunlin Li[1*], Ira Marton[1,2*], Daniel Harari[2], Maya Shemesh[2], Vyacheslav Kalchenko[3], Michal Pardo[1], Gideon Schreiber[2], Yinon Rudich[1]

[1]Department of Earth and Planetary Sciences, Weizmann Institute of Science, Rehovot 76100 Israel

[2]Department of Biomolecular Sciences, Weizmann Institute of Science, Rehovot 76100 Israel

[3]Department of Veterinary Resources, Weizmann Institute of Science, Rehovot 76100 ISRAEL.

[*] These authors equally contributed to the work

Corresponding authors: *gideon.schreiber@weizmann.ac.il*, *yinon.rudich@weizmann.ac.il*

**Seven pages**

**Three figures (Figure S1-S3)**

**Four tables (Table S1-S4)**

**One supplementary video**

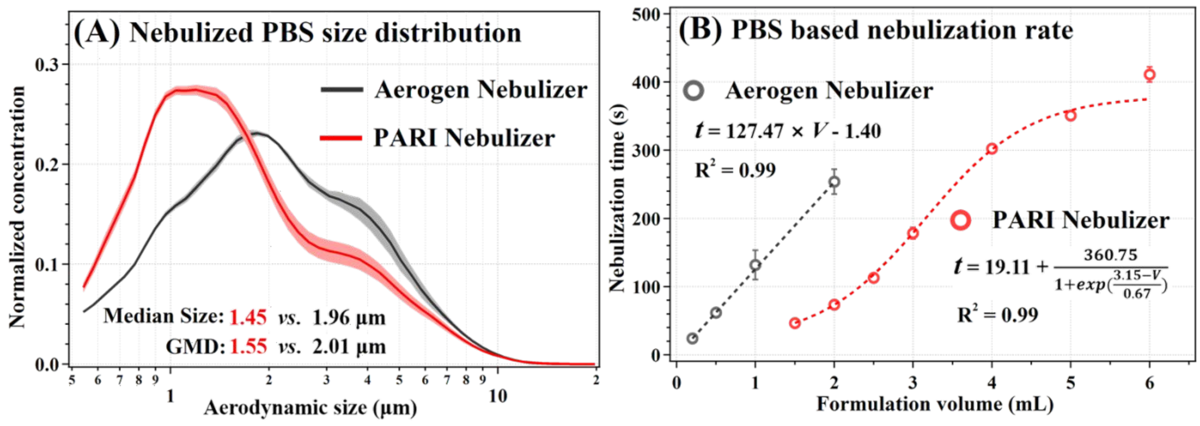


**Figure S1**. **(A)** Normalized droplet size distributions for PBS generated by the Aerogen and PARI nebulizer. **(B)** Nebulization time as a function of uploaded PBS solution volume for Aerogen and PARI nebulizer.

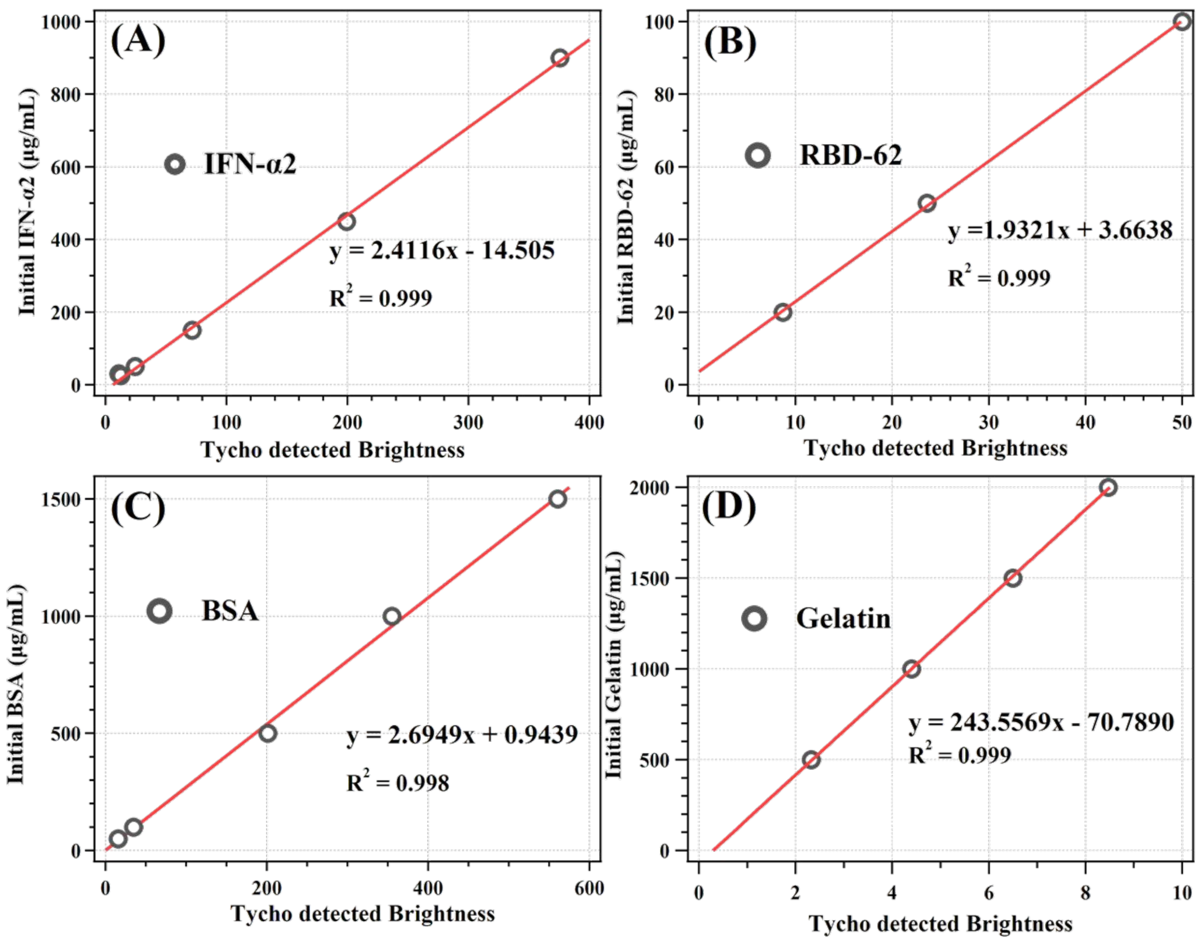


**Figure S2**. Calibration curve for protein quantification using a Tycho NT.6, relating concentration to protein signal brightness.

**Table S1**. Mediant fluorescence signals as supplementary of Figure 6.

| Test | Median fluorescence |
|---|---|
| KO2 Hela_IFN-α2 | 1574.4 |
| WT Hela_IFN-α2 | 10028 |
| KO2 Hela_IFN-β | 1438.9 |
| WT Hela_IFN-β | 13762 |

**Table S2.** Median fluorescence as supplementary to Figure 7.

| **Figure 7A** | **Median fluorescence** |
|---|---|
| HEK293 | 351.8 |
| HEK293+RBD* (plate) | 1051.9 |
| HEK293$^{ACE2}$+RBD* (plate) | 47538 |
| HEK293$^{ACE2}$+RBD* (insert) | 67283 |
| **Figure 7B** | **Median fluorescence** |
| RBD* | 61918 |
| RBD* + 100nM RBD | 2275.2 |
| RBD* + 2nM RBD-62 | 8513 |
| RBD* + 4nM RBD-62 | 1803.2 |
| RBD* + 8nM RBD-62 | 1094.8 |
| RBD* + 12.5nM RBD-62 | 673.4 |
| RBD* + 20nM RBD-62 | 628.8 |
| **Figure 7C** | **Median fluorescence** |
| 2 mg/mL Gelatin | 26142 |
| 5 µg/mL RBD-62 | 21149 |
| 20 µg/mL RBD-62 | 6287.6 |
| 100 µg/mL RBD-62 | 999.9 |
| 10 µg/mL RBD-62 + 2 mg/mL Gelatin | 2694 |
| **Figure 7D** | **Median fluorescence** |
| RBD* + BSA | 99781 |
| RBD* + 10 µg/mL RBD-62 | 7925.3 |
| RBD* + 10 µg/mL RBD-62 + 0.5 mg/mL Gelatin | 1883.7 |
| RBD* + 10 µg/mL RBD-62 + 2.0 mg/mL Gelatin | 1436 |

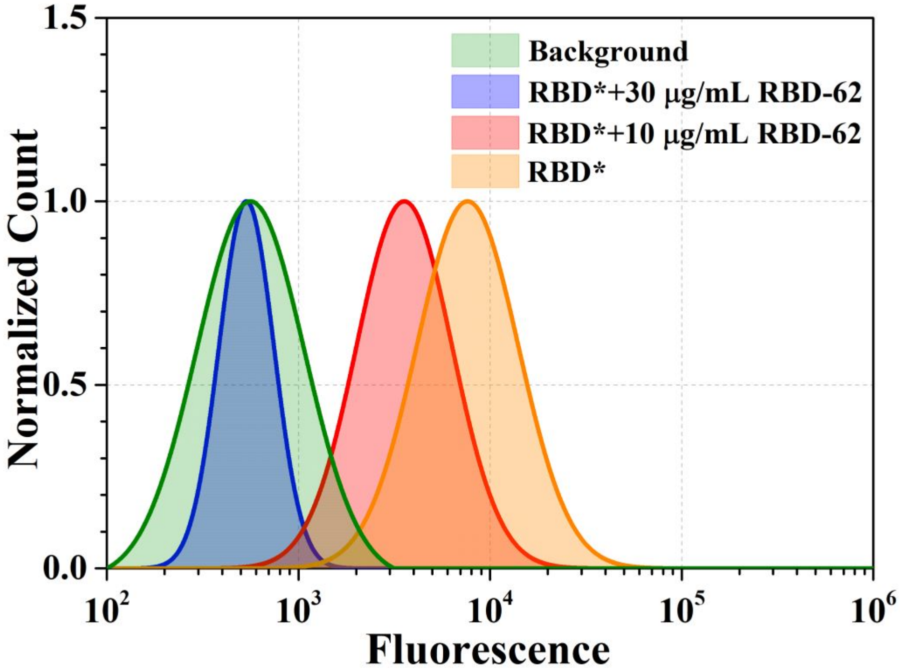


**Figure S3**. Binding competition of labeled RBD (RBD*) by non-labeled nebulized RBD-62 + 2 mg/ml Gelatin using the PARI device. The experiment is as described in Figure 7C, however, using 1 mL of RBD-62 for nebulization (of which 600 µL remains as dead volume in the device). Table blow the panel summaries median fluorescence signals for clarity.

**Table S3.** Median fluorescence as supplementary to Figure S3.

| Test | Median fluorescence |
|---|---|
| Background | 561 |
| RBD* + 30 µg/mL RBD-62 | 535 |
| RBD* + 10 µg/mL RBD-62 | 3564.4 |
| RBD* | 7625.4 |

**Table S4.** Median fluorescence of Hamster lungs after inhalation of RBD-62 labeled with CF®640R as supplementary to Figure 9A and C.

| Treatment (Fig 9A) | Median fluorescence x $10^7$ | Treatment (Fig 9C) | Median fluorescence x $10^7$ |
|---|---|---|---|
| Negative Control | 41±0.6 | Negative Control | 20±0.7 |
| 5 µg RBD-62 | 49±5.5 | 1 hr | 40±1 |
| 20 µg RBD-62 | 61±0.3 | 2 hr | 25±0.27 |
| 100 µg RBD-62 | 63±3.5 | 4 hr | 28±1 |
